# Enriched LPS Staining within the Germinal Center of a Lymph Node from an HIV-Infected Long-Term Nonprogressor but Not from Progressors

**DOI:** 10.1155/2020/7471380

**Published:** 2020-05-06

**Authors:** Lei Huang, Jianning Deng, Ren Lang, Guoyang Liao, Wei Jiang

**Affiliations:** ^1^Treatment and Research Center for Infectious Diseases, The Fifth Medical Center of Chinese PLA General Hospital, Beijing, China 100039; ^2^Department of Tuberculosis, The Fourth People's Hospital of Nanning, Nanning, Guangxi, China 530023; ^3^Department of Hepatobiliary Surgery, Beijing Chaoyang Hospital, Capital Medical University, Beijing, China 100020; ^4^Chief of No. 5 Biologicals Department, Institute of Medical Biology, Chinese Academy of Medical Sciences & Peking Union Medical College, Kuming, China 650118; ^5^Department of Microbiology and Immunology, Medical University of South Carolina, Charleston, USA 29425; ^6^Division of Infectious Diseases, Department of Medicine, Medical University of South Carolina, Charleston, USA 29425

## Abstract

An increased level of microbial translocation has been observed in HIV-infected individuals. The host response to microbial translocation is compromised in HIV-infected progressors but remains unknown in HIV-infected long-term nonprogressors (LTNPs). To evaluate microbial translocation in HIV, we assessed lipopolysaccharide (LPS) immunohistochemistry staining in lymph nodes. We found enriched bacterial LPS immunohistochemistry staining in the germinal center of a lymph node from an HIV-infected LTNP, evenly distributed from three progressors with impaired germinal center structures and rarely detected from two HIV-negative individuals. The impaired germinal center structures were consistent with collagen deposition in lymph nodes using immunohistochemistry staining. These results suggest greater immune responses against bacterial LPS translocation in LTNPs, which may reveal an important mechanism in controlling microbial translocation and disease progression in HIV LTNPs.

## 1. Introduction

HIV-infected long-term nonprogressors (LTNPs) comprise less than 1 percent of HIV-infected individuals who control HIV replication and do not progress to AIDS without medications [1]. The mechanisms of controlling disease progression in LTNPs include specific HLA types and greater HIV-specific CD8+ T cell cytotoxicity compared to progressors [2]. However, the mechanisms of host immunity to control viral replication and prevent CD4+ T cell depletion in LTNPs are not fully understood.

Chronic immune activation and inflammation are well-known hallmarks for CD4+ T cell depletion and HIV disease progression even in patients with antiretroviral therapy (ART) treatment [3]. Different therapeutic strategies targeting immune activation and inflammation (e.g., statins) have been applied to HIV-infected patients in clinic, but the effects are not clear [4]. Inflammation and chronic immune activation can be driven by microbial product translocation and residual viral effects in patients with ART treatment [5]; thus, bacterial product translocation may contribute to HIV disease progression. It remains unclear whether there is an increased level of microbial translocation in LTNPs as shown in progressors compared to healthy individuals [6, 7]. Moreover, the host immune response to microbial translocation in LTNPs remains unknown. Here we report that enriched bacterial lipopolysaccharide (LPS) immunohistochemistry staining was observed mainly in the germinal center of a lymph node from a LTNP; evenly distributed LPS was observed in lymph nodes from three progressors with impaired germinal center structures; and LPS staining was rarely observed in lymph nodes of two HIV-negative individuals.

## 2. Results and Discussion

**Figure 1:**
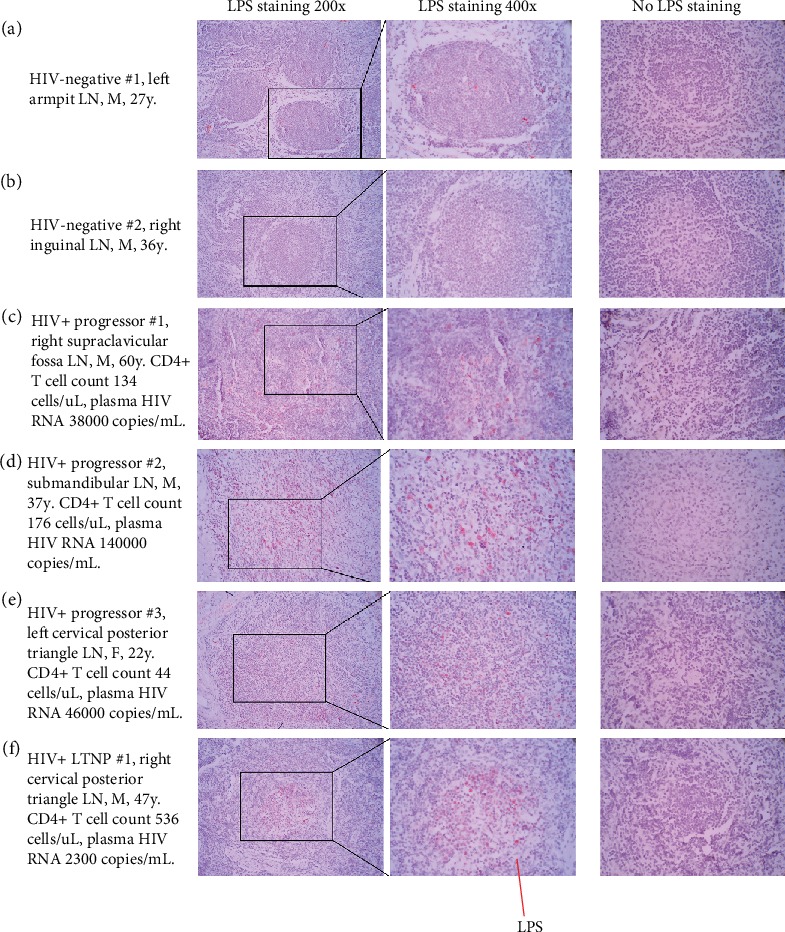
Identification of LPS staining in lymph nodes of two HIV-negative donors, three ART-naïve chronically HIV-infected progressors, and one chronically HIV-infected LTNP. Representative images of unselected lymph node (LN) sections stained for LPS-core antigen (red, 200x and 400x). The LTNP showed increased LPS infiltration within the germinal center; the progressors showed increased LPS infiltration in the LNs with impaired structures of the germinal center; LPS staining was rarely detected from the HIV-negative donors.

**Figure 2:**
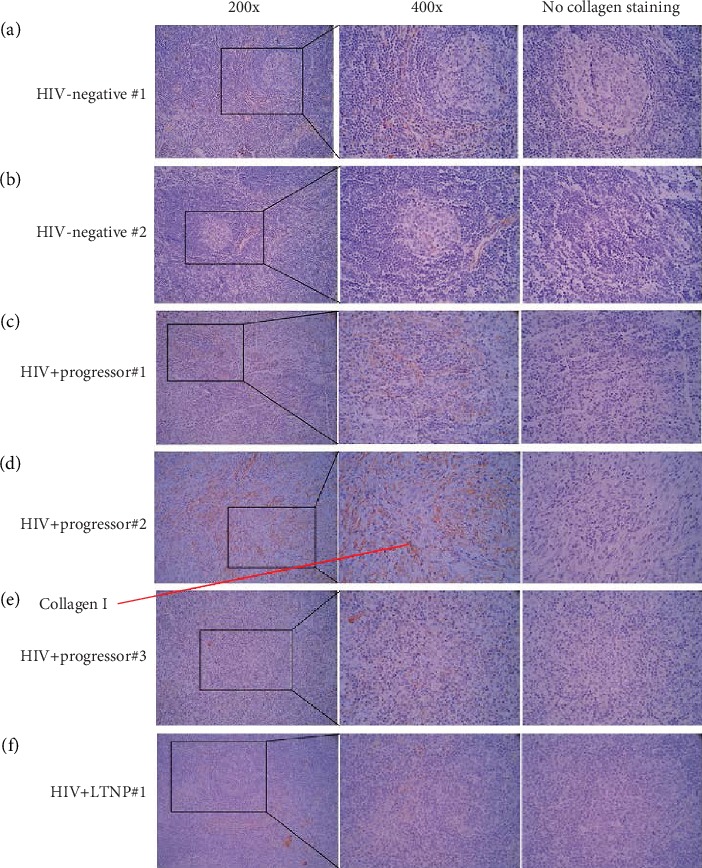
Identification of collagen I staining in lymph nodes of two HIV-negative donors, three ART-naïve chronically HIV-infected progressors, and one chronically HIV-infected LTNP. Representative images of unselected LN sections stained for collagen I antigen (red, 200x and 400x). The HIV+ progressors showed increased collagen I staining in LNs; the LTNP and HIV-negative donors showed decreased collagen I staining in the LNs compared to HIV+ progressors.

In two HIV-negative donors, LPS staining was rarely detected in their lymph nodes (Figures 1(a) and 1(b) and Table 1). Consistent with HIV-associated “leaky” gut and microbial translocation [8], LPS staining was increased in three HIV+ progressors (Figures 1(c)–1(e)) and one HIV+ LTNP (Figure 1(f)) compared to the HIV-negative donors (Figures 1(a) and 1(b)). Intriguingly, LPS was enriched and limited within the germinal center of a lymph node from the donor of LTNP, but not from the donor of progressors (Figures 1(c)–1(f)). Furthermore, the lymph nodes from HIV+ progressors exhibited impaired structures of germinal center (Figures 1(c)–1(e)), consistent with lymph node fibrosis observed in HIV+ progressors from previous studies [9]. Furthermore, to determine whether impaired germinal center structures are consistent with lymph node fibrosis, we also stained collagen I (Figure 2). Indeed, collagen deposition in the lymph node of LTNP was increased compared to those from HIV-negative control donors but decreased compared to those from HIV+ progressors (Figure 2 and Table 1). Collagen deposition was also increased in the lymphatic follicles from HIV+ progressors (e.g., progressor #1, Figure 2(c)). Therefore, the structure of lymph node from LTNP was relatively complete, and the structure of the lymph nodes of HIV+ progressors was remarkably destroyed.

Microbial translocation may play a role in chronic immune activation and inflammation, which contribute to CD4+ T cell depletion and HIV disease progression [3, 8]. However, the fundamental mechanism of chronic immune activation and inflammation and potential therapeutic targets for preventing persistent immune activation in HIV are not fully understood.

HIV-infected LTNPs are patients who are not on ART but whose CD4+ T cells remain above 500 cells/mL and who exhibit low levels of viral replication for many years (~5-10 years) [2]. This group represents less than 1% of the HIV patient population and never progresses to AIDS, the last stage of HIV disease [2]. LTNPs have reduced chronic immune activation and inflammation, as well as increased HIV-specific CD8+ T cell cytotoxicity, compared to HIV+ progressors [2]. It is not clear whether the control of disease progression in LTNPs is due to viral defects, genomic factors, or host factors. Nonetheless, LTNPs provide an excellent model to study the mechanism of the control of HIV disease progression in the host without ART treatment.

In the current study, LPS was enriched and limited in the germinal center of the lymph node from the HIV+ LTNP donor but not from the HIV+ progressors. LPS is thought as nonprotein antigen and may directly induce immune responses through Toll-like receptor 4, which is not necessary through antigen presenting and processing by antigen-presenting cells [10]. However, immune cells from LTNPs efficiently deliver LPS to the germinal center of the lymph nodes, which may result in stronger T and B cell immune responses against LPS. In addition, differences in TLR4 expression and TLR4 signaling pathway may account for the difference in host immunity against LPS in LTNPs compared to HIV+ progressors. These potential mechanisms may contribute to reduced levels of inflammation and immune activation, as well as nonprogression to AIDS in LTNPs, and deserve further investigations.

## 3. Experimental Procedures

### 3.1. Study Subjects

Six subjects were recruited for the current study: two HIV-negative subjects, three HIV+ progressors (ART-naïve and plasma HIV RNA >10,000 copies/mL), and one HIV+ LTNP. Their clinical characteristics are shown in Figure 1. The HIV+ LTNP was an HIV-infected patient who maintains low levels of plasma HIV RNA (<5000 copies/mL) and peripheral CD4+ T cell counts above 500 cells/*μ*L without ART treatment for 7 years [11]. This study was approved by the institutional review board from the Fourth People's Hospital of Nanning (Nanning, China) and the Fifth Medical Center of Chinese PLA General Hospital (Beijing, China). All participants provided written informed consents.

### 3.2. Processing of Human Lymph Nodes

Fresh human lymph nodes were obtained from an HIV-infected LTNP, three progressors, and two HIV-negative individuals who had lymph node pathological enlargements. These lymph node biopsies were fixed in formalin and embedded in paraffin for immunohistochemical staining.

### 3.3. Immunohistochemical Staining of LPS

The tissues of lymph nodes were LPS stained by immunohistochemical techniques, as described in a previous study [12]. Briefly, paraffin was removed from paraffin sections by being roasted at 65°C for 20-30 min and immersed in three xylene jars for 10 min each. Xylene was removed as sections were immersed in dehydrated alcohol for 5 min three times. These sections were further immersed in 95% ethanol for 3 min twice, and in 90%, 85%, and 80% ethanol for 2 min each. Sections were then rinsed with tap water for 3 min twice and with distilled water for 3 min twice. Antigen was retrieved in the citrate buffer (pH 6.0) for 20 min in a microwave oven. Sections were cooled at room temperature and rinsed by tap water twice. Then sections were soaked in 2% H_2_O_2_-methanol for 30 min at room temperature and rinsed by tap water for 3 min twice, distilled water for 3 min three times, and PBS (pH 7.4) for 3 min twice. Subsequently, sections were incubated with fetal bovine serum (FBS, Fisher Scientific, Hampton, NH, USA) at room temperature for 25 min. FBS was washed out, and the mouse anti-human LPS-core antibody (Cat. no. HM6011, RRID: AB_2750644; Hycult Biotech, Inc., Wayne, PA, USA) was added (1 : 200-250) to each section and incubated overnight. PBS was used to rinse sections for 5 min three times, and the secondary antibody (Cat. no. ab205719, RRID: AB_2755049; 1 : 10,000; Abcam, Cambridge, UK) was added and incubated at 37°C for 30 min. Sections were rinsed in PBS for 5 min three times and incubated with AEC at room temperature without light for 15 min. Sections were rinsed in distilled water for 2 min twice. Sections were counterstained with hematoxylin, dehydrated, cleared, and mounted with neutral gums. The negative controls were carried out with the same steps as described above, but the antibody for LPS was replaced by PBS. All stained slides were observed at a magnification of 200x or 400x, using a light microscope (Olympus CX31; Olympus Corporation, Tokyo, Japan), and were blindly evaluated by a pathologist. Fluorescence intensity analysis was performed using the ImageJ software (ImageJ, Bethesda, USA) as described previously [13].

### 3.4. Immunohistochemical Staining of Collagen I

Immunohistochemistry protocols of collagen I staining process were similar to LPS staining. The main differences in the experiment were as follows: antigen retrieval was performed using EDTA buffer (pH 8.5); endogenous peroxidase was removed using 3% H_2_O_2_-methanol solution; DAB was used for staining; and the positive result was in red. The mouse anti-human collagen I antibody was purchased from the Abcam company (Cat. no. ab88147, RRID: AB_2081873; 1 : 100; Abcam, Cambridge, UK). Stained sections were randomly selected in each donor for analysis of 5 visual fields of a magnification of 400x to detect the optical density value with the Image-Pro Plus software 6.0 for collagen I, described from our previous study [13]. The integrated optical density (IOD) of each visual field was analyzed using the SPSS software 17.0. The summarized densities are shown in Table 1.

## Figures and Tables

**Table 1:** 

	Mean ± SD (LPS)	Mean ± SD (collagen I)
HIV-negative #1	586 ± 160	4817.56 ± 4190.63
HIV-negative #2	8.7 ± 9.1	2519.05 ± 1619.79
HIV+ progressor #1	3902 ± 2166	11665.71 ± 6021.23
HIV+ progressor #2	9518 ± 2568	17343.05 ± 7313.52
HIV+ progressor #3	2490 ± 983	16156.88 ± 5086.77
HIV+ LTNP #1	2273 ± 1659	8781.22 ± 6873.75

## Data Availability

The data used to support the findings of this study are available from the corresponding author upon request.

## Abbreviations

LTNP: Long-term nonprogressor
LN: Lymph node
ART: Antiretroviral therapy
LPS: Lipopolysaccharide.
